# Autotransplantation of mature and immature third molars in 23 Chinese patients: a clinical and radiological follow-up study

**DOI:** 10.1186/s12903-017-0468-0

**Published:** 2017-12-28

**Authors:** Haozhe Tang, Zhengyan Shen, Minhong Hou, Ligeng Wu

**Affiliations:** 1 Stomatology Hospital of Enjoyment, Tianjin, China; 2 Department of Endodontics, School of Stomatology, Tianjin Medical University, Tianjin, China; 3 Department of Stomatology, Tianjin 4th Centre Hospital, Tianjin, China

**Keywords:** Mature tooth, Parodontium coalescence, Premature tooth, Tooth autotransplantation

## Abstract

**Background:**

We investigated the clinical and radiographic outcomes of autotransplanted teeth over a follow-up period of 2 to 8 years, and summarize the findings of other relevant studies with regard to the primary factors that influence a good prognosis in such patients.

**Methods:**

Twenty-three patients (6 men, 17 women) who attended Tanggu Stomatological Hospital, Tianjin, China, from 2008 through 2013, were included in the study. These patients presented with a variety of dental problems, including tooth loss, residual crowns, missing first or second molars, dental trauma, tooth fracture, and unrestored teeth. A total of 26 third molars, including 2 immature molars, were autotransplanted in these patients. The success rate of autotransplantation was assessed on the basis of clinical and radiographic outcomes after follow-up periods ranging from 2 to 8 years.

**Results:**

Clinical examination revealed stability of all 26 transplanted teeth, with satisfactory masticatory function and no patient discomfort. Radiographic examination revealed normal periapical tissues and an intact lamina dura surrounding the tooth root, indicating adequate healing of periodontal tissues.

**Conclusions:**

Autotransplantation achieved good results in the Chinese sample population investigated, and was associated with an excellent prognosis. Rigorous case selection, adequate protection of the periodontal ligament, and proper oral hygiene contribute significantly to the long-term success of the procedure.

## Background

Dental autotransplantation refers to the transplantation of a tooth from one site to another in the same individual. Autotransplantation of immature third molars as a replacement for decayed first molars was first reported in the 1950s [1]. The donor tooth can be an embedded, impacted, or erupted tooth, and the recipient site can be a previous extraction site, a surgically prepared socket, or the site of a congenitally missing tooth [2–4]. The most common donor teeth used in clinical practice are third molars [5–7], premolars [8, 9], impacted canines [10], and supernumerary teeth [11]. Both mandibular and maxillary teeth have been successfully used as donor teeth, and teeth have also been successfully autotransplanted into mandibular and maxillary sites. Mandibular donor teeth appear to exhibit the same cumulative survival rate as maxillary donor teeth [12].

Many previous studies have demonstrated that autotransplantation is a valid treatment strategy in various circumstances [3–11]. For example, viable but nonfunctional teeth, such as third molars or malpositioned teeth, can be moved to the extraction sites of nonrestorable first or second molars. Furthermore, autotransplantation is considered to be the only acceptable treatment in cases of severe canine ectopia that is unresponsive to surgical exposure and orthodontic realignment [3]. In patients undergoing orthodontic treatment requiring extraction, the premolars destined for extraction can be transplanted to sites of agenesis or anterior teeth that cannot be restored.

Currently, tooth implantation is a widely used strategy for the replacement of nonrestorable teeth. Compared with artificial implants, autotransplantation often entails a shorter treatment duration and lower cost, and in many cases, there are additional advantages [13–16]. Periodontal reattachment is possible with autotransplanted teeth, and these teeth can tolerate orthodontic movement. Autotransplantation also preserves proprioception in the periodontal ligament (PDL), resulting in appropriate phonetics and occlusion; moreover, alveolar bone can regenerate from the stem cells present in the PDLs of autotransplanted teeth and can maintain itself thereafter. Furthermore, autotransplantation improves esthetics and maintains normal gingival growth.

The reported success rates of autotransplantation vary, and immature donor teeth are reportedly associated with better outcomes than mature donor teeth. Tsukiboshi [17] reported an 82% success rate in a 6-year follow-up study of 250 cases. Lundberg and Isaksson [18] reported a 94% success rate for donor teeth with incompletely formed roots and an 84% success rate for donor teeth with completely formed roots. Majare et al. [19] reported an 82% success rate in a 4-year follow-up study of 50 autotransplanted teeth with completely formed roots.

Based on the aforementioned studies, third molars could be transplanted to replace nonrestorable teeth, and parodontium coalescence was regarded as an ideal mode of healing that could result in the transplanted teeth achieving satisfactory masticatory function. However, differences in technique, operator experience, surgical instruments, and different geographical locations have yielded different results [17–19]. Thus, we conducted this study in China in order to study the applicability of autotransplantation in China, and investigate the factors that may influence prognoses and success rates.

## Methods

This study was approved by the Stomatology Hospital of Enjoyment, Tianjin, China (application/approval number TSHEMEC20080410), and informed consent was obtained from all participants. Patients were only invited to be included in this study if they exhibited satisfactory oral hygiene, good compliance, were in good health, had ideal donor teeth for the targeted recipient sites, and if their third molars were healthy. Prosthetic implantation had been suggested to the patients included in the study, but they were hesitant due to the high cost associated with the procedure and desired to undergo autotransplantation instead. From 2008 to 2013, we have performed approximately 100 autotransplantations. However, complete clinical data were only available in 23 of these cases, and in this retrospective study we only analyzed those 23 cases.

In these 23 patients (6 men and 17 women), aged 18–42 years (mean age: 29.6 years) who visited the Stomatology Hospital of Enjoyment, Tianjin, China, from 2008 through 2013, 26 third molars were autotransplanted, including 2 with incompletely formed roots (Table 1). The indications for transplantation included severe caries (*n* = 18), vertical root fracture (*n* = 1), trauma (*n* = 1), and missing teeth (*n* = 6). All donor teeth were third molars, and the recipient sites were first or second molar sites. In one patient, the recipient site was in the anterior region (Table 2): the right lateral maxillary incisor was replaced with the right lateral maxillary third molar, as the right lateral maxillary third molar was a microdontia. The design of this study is shown in Table 3.

**Table 1:** Patient Gender and Age at the Time of Surgery

	Sex		
Age	Male	Female	Sum
10–19	1	0	1
20–29	1	8	9
30–39	4	8	12
40–49	0	1	1
Sum	6	17	23

**Table 2:** The Distribution of Autotransplanted Teeth and the Recipient Site

Recipient site									
Donor teeth	#16	#11	#26	#27	#37	#36	#47	#47	Sum
#18	3	1		1	1		2	1	8
#28	1					5			6
#38		1	1		4	1			7
#48							2	3	5
Sum	4	2	1	1	5	6	4	4	26

The numeration of teeth was according to FDI (Federation Dentaire International system)

**Table 3:** The predictors and design about this study

	Number of transplanted teeth
Stage of donor tooth root development	
6	2
7	24
Indications for transplantation	
Caries	18
Root fracture	1
Trauma	1
Missing teeth	6
Preparation of recipient sites	
Low-speed round bur	12
Planter	6
Small preparation	8
Storing of the donor tooth	
physiologic saline solution	24
Returned to primary sockets	2
Fixation method	
Sutures	9
Wires	17
Time of root canal treatment	
During surgery	1
2 weeks later	2
4 weeks later	23

Each donor tooth was required to meet several selection criteria. It had to be a nonfunctional tooth, such as a third molar. A smooth, conical, single root was considered optimal, and the shape of the donor root was required to fit well into the recipient site. The degree of tooth root development was required to be between stages 4 and 5 [20–22]. In the present study, all 26 donor teeth were nonfunctional third molars. Twenty were strong, conical, single-rooted teeth and 6 were multi-rooted teeth.

Teeth with attachment loss covering more than a third of the root length, a complicated root shape that could increase the susceptibility of the tooth to damage during extraction, a root shape that would not fit well into the recipient site, a complicated root canal system, or a rough root surface with several enamel projections were excluded.

Presurgical evaluations included the following. A general health history and assessment, including the patient’s age, overall dental hygiene (estimated based on plaque index and gingival index; oral hygiene instructions for patients were given at each visit), and history of compliance with treatment and follow-up were obtained. Additionally, radiographic evaluation of the potential donor tooth, including the root shape, stage of root development, and the condition of the root canal, and assessment of the recipient site was performed. The distance between the two adjacent teeth, alveolar bone thickness, and the distance from the root surface of the donor tooth to the alveolar bone wall when the donor tooth is placed into the recipient site were determined. In terms of the distance from the root surface of the donor tooth to the alveolar bone wall, we considered 0.5–1.0 mm, measured by cone-beam computed tomography (CBCT), as optimal, based on our several years of clinical experience. However, not all patients underwent CBCT examination, as a CBCT machine was not available during the initial stages of the study. Of the 18 patients who underwent CBCT examinations, the minimum distance was 0.5 mm. Surgical risk was determined in all patients, including consideration of the positional relationship between the maxillary molars and the maxillary sinus, and between the mandibular third molars and the mandibular canal.

The surgical procedures for autotransplantation were based on the protocols and techniques described by Andreasen et al. [20], Tsukiboshi [17], and others [23–25]. After obtaining informed consent from the patients, antisepsis was performed using an iodine swab followed by alcohol-soaked cotton to disinfect both the donor tooth and the gingiva at the recipient site. Local anesthesia was induced by nerve block using 1–2% lidocaine containing 1:200,000 adrenaline (Jinyao, Tianjin, China). Block anesthesia of the inferior alveolar nerve was used for mandibular sites, and block anesthesia of the posterior superior alveolar nerve was used for maxillary sites. The extent of the block anesthesia was adequate for both extraction and transplantation.

We used 2 different surgical procedures, depending on whether the recipient site held a diseased tooth (*n* = 20) or was missing a tooth (*n* = 6). Diseased teeth at recipient sites were extracted. Of the 20 diseased teeth, 14 had multiple roots and required split-root surgery, which was performed using a high-speed fissure bur (FG-700, Meisheng Corporation, Shanghai, China). The recipient site was thoroughly curetted to prevent infection. For 12 of the 20 teeth requiring extraction, the recipient site was modified with a low-speed round bur (FG-3, Mengsheng Corporation, Shanghai, China) to achieve a good fit with the donor tooth. When the recipient site was missing a tooth, the socket was surgically prepared using implant burs (INTRAsurg 1000, KaVo Dental GmbH, Leipzig, Germany) to match the shape of the donor tooth.

**Fig. 1:**
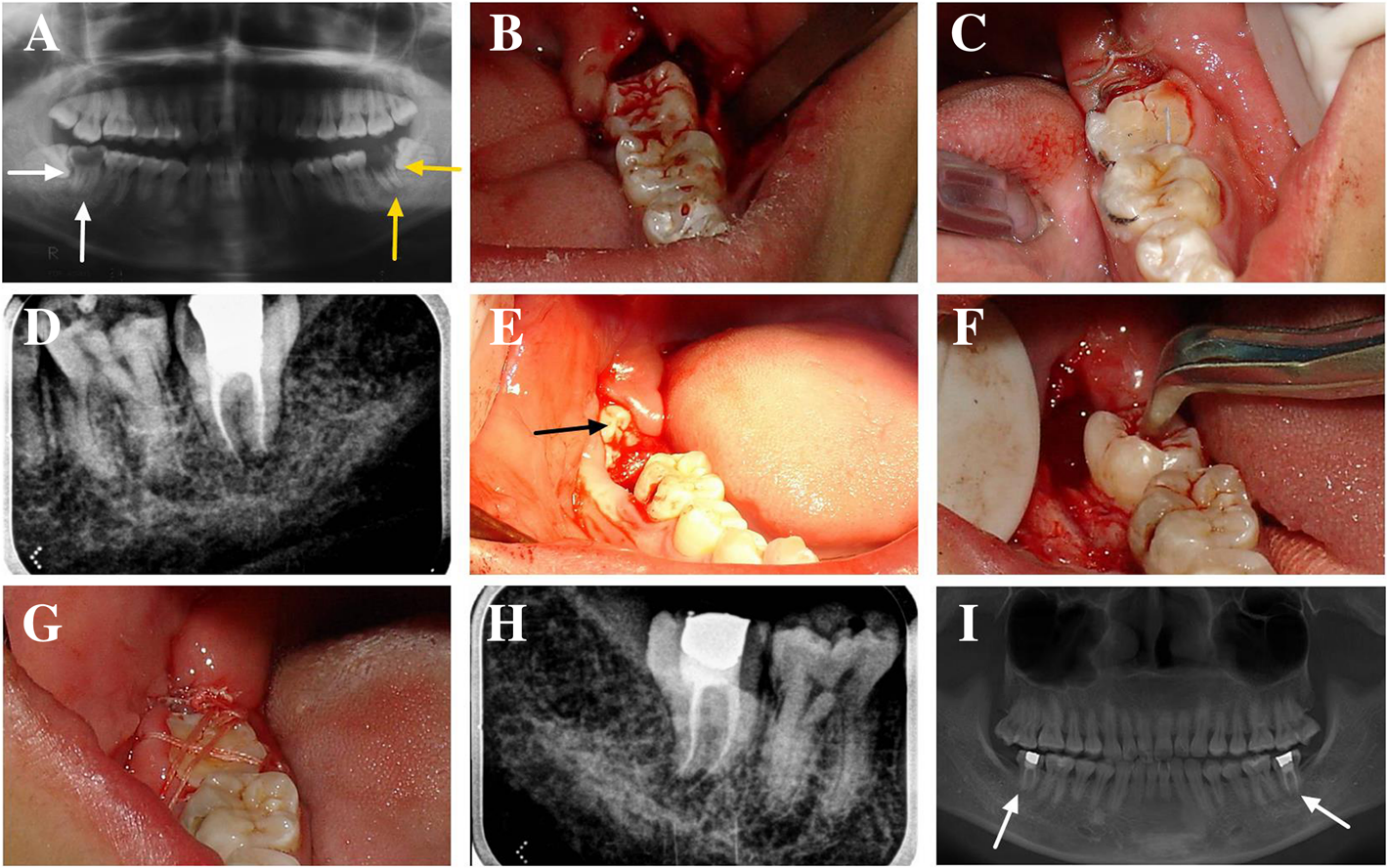
Course of treatment and long-term follow-up in a 24-year-old patient who had 2 third molars transplanted. The #38 tooth replaced the #37 tooth, and the #48 tooth replaced the #47 tooth. **a** A panoramic radiograph revealed severe caries of the left and right mandibular second molars (#37, #47). Radiolucent areas were visible at the apices of the #37 and the #47 teeth. Neither could be restored, and both were extracted. **b** The #38 tooth was transplanted into the #37 position after being rotated 180°. **c** The #38 tooth was fixed with wire after occlusal adjustment. **d** An intraoral radiograph of the #38 tooth immediately after endodontic treatment 4 weeks after transplantation. **e** An intraoral photograph of the #48 tooth before transplantation. **f** The #48 tooth was transplanted into the #47 position after being rotated 180°. **g** The #48 tooth was fixed via suturing because the #48 tooth extended too far. After occlusal adjustment, the edge of the #48 tooth was at the level of the gingiva, and it was difficult to fix with wire. **h** An intraoral radiograph of the #48 tooth immediately after endodontic treatment 4 weeks after transplantation. **i** Panoramic radiograph after 7 years of follow-up. The radiolucent areas at the apices of the #38 and the #48 teeth have disappeared

Extraction of the donor teeth was minimally invasive, and was performed using dental forceps (Armamentarium Corporation, Shanghai, China) in 23 cases and a slender dental lever (Armamentarium Corporation, Shanghai, China) in 3 cases. In all 26 cases, the donor tooth was rotated 180° in the recipient socket to achieve an optimal fit, followed by fixation and occlusal adjustment. Fixation was achieved with sutures after occlusal adjustment in 9 cases, while in the remaining 17 cases it was achieved using a flexible single-end steel splint before occlusal adjustment. Lastly, the soft tissue flap was trimmed and sutured in place (Fig. 1).

**Fig. 2:**
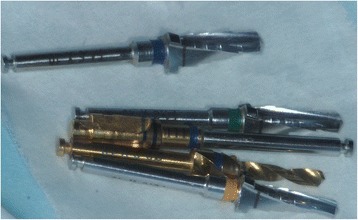
Photograph of the guide plates and implant burs during surgery. We used 3 guide plates in 3 directions

For 1 patient, we used an Object Eden500V 3D printer (Stratasys, Rehovot, Israel) to create a model of the donor tooth from digital radiographic images obtained prior to the transplant procedure. We also used a guide plate in combination with an implant bur (Fig. 2). The use of 3D printed guides is relatively common in some countries [26], but they are not commonly used in China, and particularly not in combination with a guide plate in autotransplantation. Thus, we randomly selected a case in which to perform the procedure, with the aim of utilizing it in future clinical practice if the results were satisfactory.

The PDLs of the donor teeth were preserved by using a comparatively un-invasive technique during extraction, with the donor teeth stored appropriately after extraction and before transplantation (24 teeth were stored in physiological saline solution and 2 were temporarily returned to their primary sockets). Donor teeth were gently placed into the recipient sites, without applying pressure, and avoiding mechanical damage to the PDL attached to the donor tooth. The PDL at the recipient site was preserved using split-root surgery during the extraction of multirooted teeth, wherein each root was individually removed. Lastly, an extraoral time of less than 18 min was maintained to preserve the PDLs of all 26 teeth. Numerous measures were taken to minimize extraoral time, including involvement of an experienced surgeon and operator, multidisciplinary cooperation, and sufficient preoperative preparation.

We utilized single-visit endodontic treatment. The step-back technique was used as the root canal preparation technique. The working length was determined via an apex locator and X-ray. The initial apical file was a #15 (ISO) K file. For the 2 teeth with incompletely formed apices, the master file was a #40 (ISO) K file, and for teeth with completely formed apices, the master file was a #30 (ISO) K file. In root canal irrigation, 3% hydrogen peroxide solution, 0.9% normal saline (NS), and 0.25% sodium hypochlorite solution were used. Gutta-percha points were used as obturation material, and calcium hydroxide paste was used as a sealer. The lateral condensation technique was used for obturation.

In the immediate postoperative period, patients were prescribed the following antibiotic regimen: roxithromycin (Haining, Nanjing, China) 150 mg once a day for 5–7 days and ornidazole (Haining, Nanjing, China) 500 mg twice a day for 5–7 days. Splints were removed after a week, and root canal treatment was performed 2–4 weeks after surgery.

We used the criteria described by Tsukiboshi [3] and Lee [4] to assess the outcomes of tooth transplantation. Successful autotransplantation was determined based on the following criteria: Normal pocket depth, gingival contour, and gingival color on clinical examination after fixing the tooth in its socket; satisfactory masticatory function with no chewing discomfort; absence of radiographic evidence of residual inflammation or pathology; and presence of a normal lamina dura. The evaluations included gingival index, probing depth, clinical attachment level, mobility, restoration of masticatory function, and radiographic examination.

## Results

Patients were followed up at 1 week, 2 weeks, 4 weeks, 2 months, 4 months, 6 months, 1 year, and 2 years. Some of the cases were followed up for as long as 8 years. All 23 patients were compliant with their follow-ups. The results of the evaluations are shown in Tables 4 and 5.

**Table 4:** The number of cases for the results of clinical examination at baseline and follow-up period

	Baseline	Follow-up period							
		One week	Two week	Four week	Two month	Four month	Six month	One year	Two year
Gingival index									
0	6	0	4	8	20	26	26	26	26
1	18	7	11	18	6	0	0	0	0
2	2	19	11	0	0	0	0	0	0
Probing depth									
1 mm	0	–	–	–	2	3	3	3	3
2 mm	4	–	–	–	18	20	20	20	20
3 mm	14	–	–	–	6	3	3	3	3
4 mm	2	–	–	–	0	0	0	0	0
Mobility									
Negative	12	0	0	0	2	10	26	26	26
I	3	0	0	12	16	16	0	0	0
II	4	6	18	14	8	0	0	0	0
III	1	20	8	0	0	0	0	0	0
Restoration of masticatory function									
Satisfactory	–	–	–	4	13	20	23	26	26
Dissatisfactory	–	–	–	22	13	6	3	0	0
Radiographic examination									
Inflammation	19	19	16	14	6	2	0	0	0
Normal	7	7	10	12	20	24	26	26	26

**Table 5:** As for different predictors, the clinical result of the 26 donor teeth at one year follow-up

	Number of transplanted teeth	Gingival index	Probing depth (mm)	Mobility	Masticatory function	Radiographi--c examination
Stage of donor tooth root development						
6	2	0	2	Negative	Satisfactory	Normal
7	24	0	1,2,3	Negative	Satisfactory	Normal
Indications for transplantation						
Caries	18	0	2,3	Negative	Satisfactory	Normal
Root fracture	1	0	1	Negative	Satisfactory	Normal
Trauma	1	0	1	Negative	Satisfactory	Normal
Missing teeth	6	0	1,2	Negative	Satisfactory	Normal
Fixation method						
Sutures	9	0	2,3	Negative	Satisfactory	Normal
Wires	17	0	1,2,3	Negative	Satisfactory	Normal
Time of root canal treatment						
During surgery	1	0	2	Negative	Satisfactory	Normal
2 weeks later	2	0	2	Negative	Satisfactory	Normal
4 weeks later	23	0	1,2,3	Negative	Satisfactory	Normal

In terms of PDL healing, 2 months after transplantation, near-normal PDL and alignment of collagen fiber bundles were observed in most cases, and only 6 cases exhibited periapical periodontitis, and healing with minor residual inflammation. At 6 months after transplantation, all 26 teeth had achieved parodontium coalescence, and radiographic analysis of all 26 revealed no pathological radiolucency or tooth resorption, and a continuous periodontal space was observed around each root.

In terms of gingival restoration, there was inflammation and edema with bleeding on probing at 1 week after transplantation. There was no bleeding on probing 4 weeks later, but minor edema remained. Most of the teeth were normal at 2 months, but minor edema was present in several cases. All teeth exhibited normal gingiva 4 months later, with a good gingival contour and gingival color. At 2 months after transplantation, the probing depths of all transplanted teeth were normal.

In terms of mobility, immediately after autotransplantation the recipient sockets were wide in all 26 transplanted teeth. One week after surgery, most teeth exhibited mobility of 3°. Two weeks later, the mobility of most donor teeth was assessed as grade 2, and several were assessed as grade 3. Two months later, the mobility of donor teeth was assessed as grade 1 or 2. At 4 months, only half the teeth exhibited mobility of grade 1 and 6 months later all of these teeth demonstrated normal physical mobility.

In conjunction with the recovery of physical mobility, the periodontal condition improved and the teeth gradually began to function normally. All patients exhibited satisfactory masticatory function, and there were no complaints of pain, discomfort, or other adverse events. At the 2-year follow-up time-point, all 26 autotransplanted teeth met the success criteria, resulting in a 100% success rate. As all 26 teeth yielded the same results at the 2-year follow-up time-point, we did not perform statistical analysis.

## Discussion

Here we have reported the clinical and radiographic outcomes of autotransplanted teeth over a follow-up period of 2 to 8 years and summarize the findings of previous studies with regard to the primary factors that influence a good prognosis in these patients. Previous literature on autotransplantation reports that selection criteria [6, 27], preservation of the PDL [28], extraoral time of autotransplanted teeth [21, 29, 30], age of patients, stage of donor tooth root development [17, 20, 22, 31–34], fixation methods and duration [9, 33], and the use or nonuse of endodontic treatment after surgery [17] are primary factors influencing the prognosis of autotransplanted teeth. In the present study, we found that these 7 factors, as well as the adaptation of the autotransplanted teeth to the recipients’ sockets and the quality of root canal treatment were major prognostic factors, while patient age, fixation method and duration, and occasion for root canal treatment were not very influential factors.

Preservation of both the PDL at the recipient site and that attached to the transplanted tooth root is essential. We used different measures to preserve the PDL. Firstly, a short extraoral time was the primary factor in PDL preservation. The vitality of the PDL is reported to decrease markedly after 18 min in an extraoral environment [21, 30]. Accordingly, we utilized an extraoral time of less than 18 min for all 26 teeth.

Secondly, proper storage of donor teeth is also important. A previous study has shown that storage of teeth in physiological saline solution and saliva offers good protection for periodontal and pulp healing during the extra-alveolar period [21]. In the present study, 24 teeth were stored in physiological saline solution and 2 were temporarily returned to their primary sockets. Of the 2 donor teeth that were temporarily returned to their primary sockets during recipient site preparation, 1 was a good fit for the recipient site, which therefore did not require much preparation. The other was the aforementioned case in which we trialed the use of a 3D printer, and because we could use the 3D printed tooth to examine the fitness of the recipient site, we temporarily placed the donor tooth back in its primary socket.

Moreover, the use of a minimally invasive technique during extraction of donor teeth, gently placing donor teeth without applying pressure at the recipient site, and split-root surgery during the extraction of multirooted teeth at the recipient site were also beneficial in preserving the PDL.

With respect to adaptation of the autotransplanted tooth in the recipient socket, the distance between the transplanted tooth and the recipient socket should not be too small or too large, as confirmed by Tsukiboshi et al. [35]. However, no exact distances have been reported in the literature. In the current study, the distance of 1 mm that was used was based on our clinical experience over a period of more than 10 years. We also consulted additional surgeons, and we believe that a distance of 0.5–1.0 mm is optimal.

If the distance is too small, the bone tissue reaches the root surface in too short a time and ankylosis is likely to occur at the site of the injured PDL. If the distance is too large, the time taken for the bone tissue to reach the transplanted tooth root is prolonged, leading to extensive repair of the root surface attachment, and complicating PDL healing. When an extraction was required prior to autotransplantation, we always chose a donor tooth exhibiting maximum compatibility with the recipient site.

In our opinion, the quality of root canal treatment is critical to the success of autotransplantation. Inappropriate or incomplete treatment leads to residual inflammation in the pulp cavity or lateral pulp canals, which causes the infection to spread toward the periapical region and results in periapical disease and root canal failure. In the present study, all 26 autotransplanted teeth underwent complete root canal treatment.

Patient age, the fixation method and duration, and need for root canal treatment were not relatively critical for the success of autotransplantation. It has been reported that patients aged younger than 20 years [34] and the use of donor teeth with immature roots [17, 20, 22, 31, 32] result in higher success rates. In the present study, the patients were aged 18–42 years, with a mean age of 29.6 years. Moreover, wire fixation reportedly results in a lower success rate [33]. However, in the present study, 17 transplanted teeth were fixed with wires and nine with sutures, and all teeth remained fixed for 1 week. There was no significant difference between the two fixation methods in terms of outcome.

In terms of the need for root canal treatment, although root canal treatment is recommended at 1 to 2 weeks after surgery [17], we performed it before surgery for two teeth, during surgery for one, and at 4 weeks after surgery in 23. We recommend root canal treatment before autotransplantation surgery only if the donor tooth was a normotopic third molar and if the patient had adequate mouth opening.

There were two reasons for immediate root canal treatment of teeth with incompletely formed roots. Firstly, although teeth with incompletely formed roots may achieve healing of dental pulp and root development, obliteration of the pulp canal is very common. If the obliteration is incomplete, the residual pulp is susceptible to infection, and this infection may rapidly develop into apical periodontitis [36]. Secondly, when the donor tooth root development is at stages 4 or 5, it is more likely that healing of the dental pulp and root development will be achieved [20]. However, the donor tooth root development in our study was at stage 6, and it was very difficult to achieve healing of dental pulp. Thus, we chose to immediately root-fill the teeth with incompletely formed roots.

An Object Eden500V 3D printer was used to create a model of the donor tooth in one patient. We also used a guide plate in combination with an implant bur, which limited the range of movement within the alveolar bone and easily prevented damage to important adjacent anatomical structures. Overall, the use of the 3D model along with the guide plate significantly decreased the extraoral time, which was a total of 10 min. Moreover, it was easier to identify rough projections in the recipient socket when thin silver paper was wrapped around the 3D–printed tooth.

Autotransplantation is not a new technique, but most reports to date have been from the Occident, and from Japan [3, 4] and Korea [2, 5] in Asia, with few such reports from China. Different institutions may adhere to different concepts, and possess different technologies, experience, and surgical instruments, which may lead to different surgical outcomes. The current study has enriched the knowledge base pertaining to autotransplantation. Furthermore, the 3D printer technique trialed in the study was not new, but the use of a 3D printer in combination with a guide plate has not been reported previously. This technique may substantially facilitate protection of the PDL attached to the transplanted tooth root. Although these observations should be confirmed in a larger, well-designed study, this approach may become widely used in clinical practice.

We obtained a 100% success rate in the current study, which may be due to a number of factors. One factor was the strict selection criteria, not only for the donor teeth, but also for the recipient sockets. Another was the protection of the PDL at the recipient site and that attached to the transplanted tooth root. In addition to these factors, control of periodontal condition was also important, and oral hygiene instructions were issued to patients at each visit.

The current study had several limitations. One was the sample size, only 26 teeth in 23 patients. Another was the study design, in that it was not a strictly controlled study and uniform procedures were not used in every case; for example, the 3D printer combined with a guide plate was only used in a single case. Lastly, the follow-up periods were 2–8 years, which is a broad range. Due to these limitations, further well-designed studies including randomized controlled trials and incorporating large sample sizes are needed.

## Conclusions

The results of the current study suggest that autotransplantation is highly applicable in China, and is associated with an excellent prognosis. We believe that strict selection criteria, protection of the PDL, and proper oral hygiene contributed to the very high success rate in the current study as compared with other studies.

## Abbreviations

CBCT: Cone beam computed tomography
PDL: Periodontal ligament
